# Synergistic Effects of the ThriveCo Bumps Eraser Kit: Exfoliating Scrub and Lotion in Reducing Skin Bumps and Enhancing Hydration With Spirulina, Alpha-Hydroxy Acid, Madhuca indica Oil, Allantoin, and Hydroxyethyl Urea

**DOI:** 10.7759/cureus.92758

**Published:** 2025-09-19

**Authors:** Maheshvari N Patel, Nayan Patel, Apeksha Merja, Saurav Patnaik, Shatakshi Maulekhi

**Affiliations:** 1 Clinical Research, NovoBliss Research Private Limited, Ahmedabad, IND; 2 Pharmacology, Swaminarayan University, Kalol, IND; 3 Clinical Research Operations, NovoBliss Research Private Limited, Ahmedabad, IND; 4 Dermatology, NovoBliss Research Private Limited, Ahmedabad, IND; 5 Cosmetology, Anveya Living Private Limited, Gurgaon, IND

**Keywords:** hydration, madhuca indica, skin bumps, spirulina, strawberry legs

## Abstract

Introduction

Keratosis pilaris, commonly referred to as "strawberry legs" or "chicken skin," is a benign yet cosmetically concerning condition characterized by dry, rough, and bumpy skin, predominantly affecting females. It often causes discomfort, such as itchiness and irritation, due to impaired skin texture and hydration. The ThriveCo Bumps Eraser Kit (Beaucience India Private Limited, Faridabad, India), comprising an exfoliating scrub and smoothing body lotion, offers a comprehensive solution. The scrub enhances texture through gentle exfoliation, while the lotion hydrates and soothes irritation. This dual-action approach addresses key symptoms and promotes smoother, healthier skin.

Methods

This interventional, prospective clinical study evaluated the safety and efficacy of the Bumps Eraser Kit. Ethical approval was obtained, and informed consent was collected. Skin hydration was assessed by Corneometer® CM 825 (Courage + Khazaka Electronic GmbH, Cologne, Germany), pigmentation by Mexameter® MX 18 (Courage + Khazaka Electronic GmbH), and surface texture by VISIOSCAN® VC20 Plus (Courage + Khazaka Electronic GmbH), along with Visual Analogue Scale and digital photographs taken on Days 01, 21 (+2), and 45 (+2). Statistical analysis was performed using IBM SPSS Statistics for Windows, Version 29 (Released 2021; IBM Corp., Armonk, New York, United States) and Microsoft® Excel 2019 (Microsoft® Corp., Redmond, WA, USA), with significance set at 5%.

Results

The study showed significant improvements. Skin hydration increased by 131.75% (p < 0.0001), redness reduced by 69.30%, pigmentation decreased by 35.77%, and itchiness dropped by 92.63%. Smoothness improved by 55.00%, roughness reduced by 148.54%, and scaliness decreased by 87.09% (all p < 0.0001). No adverse effects were reported.

Conclusion

The ThriveCo Bumps Eraser Kit improves skin texture and hydration. The scrub exfoliates and offers antioxidant benefits, while the lotion nourishes with ingredients like spirulina, alpha-hydroxy acids (AHAs), Madhuca indica oil, allantoin, and hydroxyethyl urea. Together, they reduce redness, pigmentation, and irritation, making it an effective addition to daily skincare for smoother, healthier-looking skin.

## Introduction

Keratosis pilaris (KP), often referred to as chicken skin or sometimes described as strawberry legs, is a common benign dermatological condition that commonly manifests during childhood and adolescence. While rarely discussed in clinical studies, it can be a bothersome issue, particularly for females, causing dryness, itching, and rough, dry skin. The condition is characterized by clogged hair follicles and the presence of raised papules, often leading to discomfort and aesthetic concerns [1]. KP typically presents as keratotic follicular papules, most commonly found on the extensor surfaces of the upper arms, upper legs, and buttocks. Other affected areas may include the face and trunk. These small, acuminate papules, around 1 mm in size, create a stippled, gooseflesh-like appearance, which differs from cutis anserina, a piloerector response to sympathetic stimuli. The papules often contain fine, coiled, brittle hairs [2].

Skin care products are widely available and play a crucial role in health and personal care. Their moisturizing or antioxidant effects are often attributed to active ingredients such as urea, which helps maintain skin hydration and improve overall skin health [3]. Urea, a key component of the skin's natural moisturizing factor (NMF), plays a crucial role in maintaining skin hydration and integrity and is considered safe for use in cosmetics under current practices, provided it is formulated to be non-irritating and used at concentrations consistent with those outlined in the safety assessment [4]. Urea was first used in modern medicine for wound treatment due to its proteolytic and antibacterial properties. As part of the NMF, it helps maintain skin hydration and the integrity of the stratum corneum. Urea also supports skin barrier function, regulates keratinocyte gene expression, and promotes keratinocyte proliferation [5].

*Madhuca latifolia* (syn. *M. indica*) of the Sapotaceae family is a valuable medicinal and economic plant. It is known for its emollient, stimulant, heating, demulcent, and astringent properties, and is commonly used to treat swelling, itching, and skin diseases. The plant contains fatty acids, sapogenins, sugars, triterpenoids, steroids, saponins, flavonoids, and glycosides, contributing to its therapeutic benefits [6].

Allantoin is widely used in the cosmetic industry, particularly in body creams, lotions, and shower gels, as an emollient ingredient. It helps to soothe and protect the skin, alleviating the irritation caused by exfoliating agents [7]. Despite its widespread use in both pharmaceutical and cosmetic products for topical application, its effectiveness in promoting skin healing and regeneration remains well-regarded. Allantoin is also utilized as a keratolytic agent in the treatment of itchy skin [8]. It enhances smoothness by promoting exfoliation. Its moisturizing properties make it an ideal ingredient in skincare products designed to hydrate and nourish the skin, particularly for individuals with dry, rough, or damaged skin [9].

*Spirulina platensis* is rich in phytochemicals such as proteins, carbohydrates, flavonoids, phenols, terpenoids, and steroids, which contribute to its antioxidant properties. Recognized as a valuable source of antioxidant compounds, spirulina has shown potential in neutralizing free radicals and protecting cells from oxidative damage [10]. It promotes a healthy environment for skin cells and structure, supporting its anti-aging and photoprotective benefits. Additionally, its antimicrobial properties contribute to the management of acne, further enhancing its skincare potential [11].

Alpha-hydroxy acids (AHAs) are naturally occurring organic acids, including glycolic, citric, malic, tartaric, and lactic acid, found in various foods and milk sugars [12]. AHAs are natural, physiological substances that help modulate skin keratinization and stimulate the biosynthesis of dermal components. Due to these effects, AHAs are therapeutically beneficial for treating dry and rough skin, acne, rosacea, warts, eczema, and age-related skin changes, including wrinkles and photoaging. They are also advantageous for sensitive or compromised skin, aiding in the prevention of oxidative damage caused by UV radiation [13].

This study aimed to evaluate the effectiveness of the ThriveCo Bumps Eraser Kit (Beaucience India Private Limited, Faridabad, India), which includes the ThriveCo Bumps Eraser Exfoliating Scrub and ThriveCo Smoothing Body Lotion. The primary objective was to assess improvement in skin hydration, redness, pigmentation, and itchiness from baseline to post-treatment, with the goal of determining the product’s impact on skin health and appearance. The secondary objectives focused on changes in skin texture (smoothness, roughness, and scaliness) before and after usage. Digital photographs were analyzed for visual improvements, and treatment perception was assessed. A subjective consumer perception survey was also conducted to evaluate user satisfaction, offering a comprehensive analysis of both clinical efficacy and participant-reported outcomes.

## Materials and methods

Ethical conduct of the study

The clinical investigation, including the Informed Consent Document (ICD), was reviewed and approved by the ACEAS Independent Ethics Committee, which is registered with the Central Drugs Standard Control Organization (CDSCO; registration no. ECR/281/Indt/GJ/2017/RR-21) and the Office for Human Research Protections (OHRP), U.S. Department of Health and Human Services (DHHS; registration no. IRB00011046). The study protocol (Ver#1.0), English and Gujarati ICD (Ver#1.0), CRF (Ver#2.0), and other relevant documents received approval from the ACEAS Independent Ethics Committee on December 5, 2023, prior to the commencement of the study.

The study adhered to the established standard operating procedures (SOPs), the study protocol and its amendments, Good Clinical Practice (GCP) guidelines for Clinical Research in India (2005), the New Drugs and Clinical Trials Rules (2019), the International Council for Harmonisation (ICH) E6 (R2) on "Good Clinical Practice," the Indian Council of Medical Research (ICMR) National Ethical Guidelines for Biomedical and Health Research Involving Human Participants (2017), and the Declaration of Helsinki (Brazil, October 2013). This clinical trial has been registered with the Clinical Trial Registry of India (CTRI) under the trial registration number CTRI/2024/01/061138 (registered on: 05/01/2024).

Study design

This was an interventional, prospective, clinical safety and efficacy study of Bumps Eraser Kit (Bumps Eraser Exfoliating Scrub and Smoothing Body Lotion) conducted in healthy females experiencing KP or strawberry legs, or extremely dry skin. The study was conducted at Contract Research Organization - NovoBliss Research Private Limited, Ahmedabad, India, over a period of 45 days, with a total of 32 healthy adult female subjects enrolled, and 30 subjects completed the study.

The primary objectives of the study were to evaluate the effectiveness of the test kit in improving skin hydration, reducing skin redness and pigmentation, and alleviating skin itchiness. The secondary objectives of the study were to evaluate the effectiveness of the test kit in improving skin texture, including smoothness, roughness, and scaliness, by comparing baseline and post-treatment assessments.

This study included healthy, non-lactating, non-pregnant females aged 18 to 60 years, with bumps, KP, strawberry legs, or extremely dry skin. Females of childbearing potential were required to have a negative urine pregnancy test and maintain stable contraceptive use for at least six weeks prior to the study. Exclusion criteria included active skin infections, history of dermatological conditions, known allergies to test kit ingredients, use of systemic therapies (e.g., antibiotics, retinoids, oral steroids) within four weeks prior, use of topical retinoids within two weeks, pregnancy or breastfeeding, history of scar treatments, substance abuse, chronic illnesses affecting the skin, or participation in similar trials within the past month. Subjects were required to agree not to use any medicated treatments for bumpy skin other than the study treatment.

Subjects were contacted by phone to confirm their agreement and instructed not to use any skincare products on the day of the study visit. The study included three scheduled visits: Visit 01 (Day 01) for screening and enrollment, Visit 02 (Day 21 +2 days) for test kit usage and evaluations, and Visit 03 (Day 45 +2 days) for final evaluations and study completion. Efficacy assessments, including skin hydration, appearance, and itchiness, were conducted using the Corneometer® CM 825 (Courage + Khazaka Electronic GmbH, Cologne, Germany), Mexameter® MX 18 (Courage + Khazaka Electronic GmbH), VISIOSCAN® VC20 Plus (Courage + Khazaka Electronic GmbH), Visual Analogue Scale (VAS), and digital photographs on Days 01, 21 (+2 days), and 45 (+2 days).

Details about test products

The ThriveCo Bumps Eraser Kit (by Anveya Living Private Limited) includes two test products: the ThriveCo Bumps Eraser Exfoliating Scrub and the ThriveCo Smoothing Body Lotion. The Bumps Eraser Exfoliating Scrub was applied by wetting the skin, massaging the test product in a circular motion for three to four minutes, and then washing it off with water. This was followed by the application of the Smoothing Body Lotion, which is massaged into clean, dry skin all over the body or on problem areas until fully absorbed. The scrub was recommended for use three times a week, while the body lotion should be applied daily. Both products are for topical use.

Statistical analysis

Continuous variables were analyzed using descriptive statistics, including the number of observations (N), mean, standard deviation (SD), median, as well as the minimum and maximum values. Categorical variables were summarized by frequency and percentage, with graphical representations included where appropriate. Adverse events (AEs) were summarized in terms of both number and percentage. Statistical analyses were conducted using IBM SPSS Statistics for Windows, Version 29 (Released 2021; IBM Corp., Armonk, New York, United States) and Microsoft® Excel 2019 (Microsoft® Corp., Redmond, WA, USA), with a significance level set at 5%. Subjects who withdrew from the study were not included in the statistical analysis.

Sample size determination

The sample size for this study was determined based on insights from a thorough literature review, providing guidance on typical enrolment numbers for similar research. This approach ensured a balance between feasibility and scientific rigor, with a target of 30 completed subjects to account for potential dropouts. A total of 32 subjects were enrolled, and 30 successfully completed the study, ensuring data robustness and reliability. The sample size was carefully selected to generate meaningful outcomes while maintaining a 95% confidence interval, reinforcing the validity and reliability of the study’s findings.

## Results

Evaluation

Demographic and Other Baseline Characteristics

**Figure 1:**
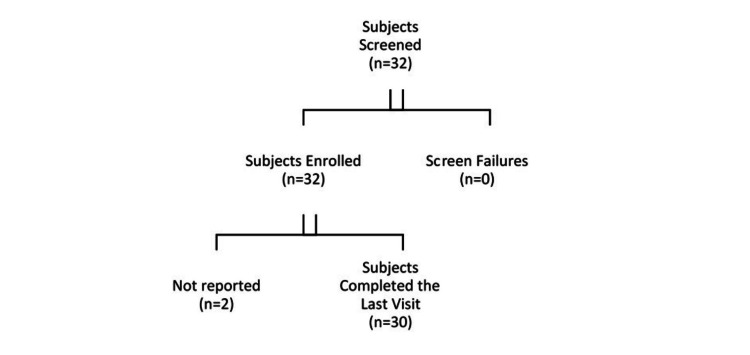
Demographic disposition of participants

In this study, there were 32 females enrolled; all 32 (100.00%) subjects received the test kit. Out of 32 subjects, 30 subjects completed the study (Figure 1).

Primary efficacy endpoints

Skin Hydration Assessed by the Corneometer® CM 825

**Figure 2:**
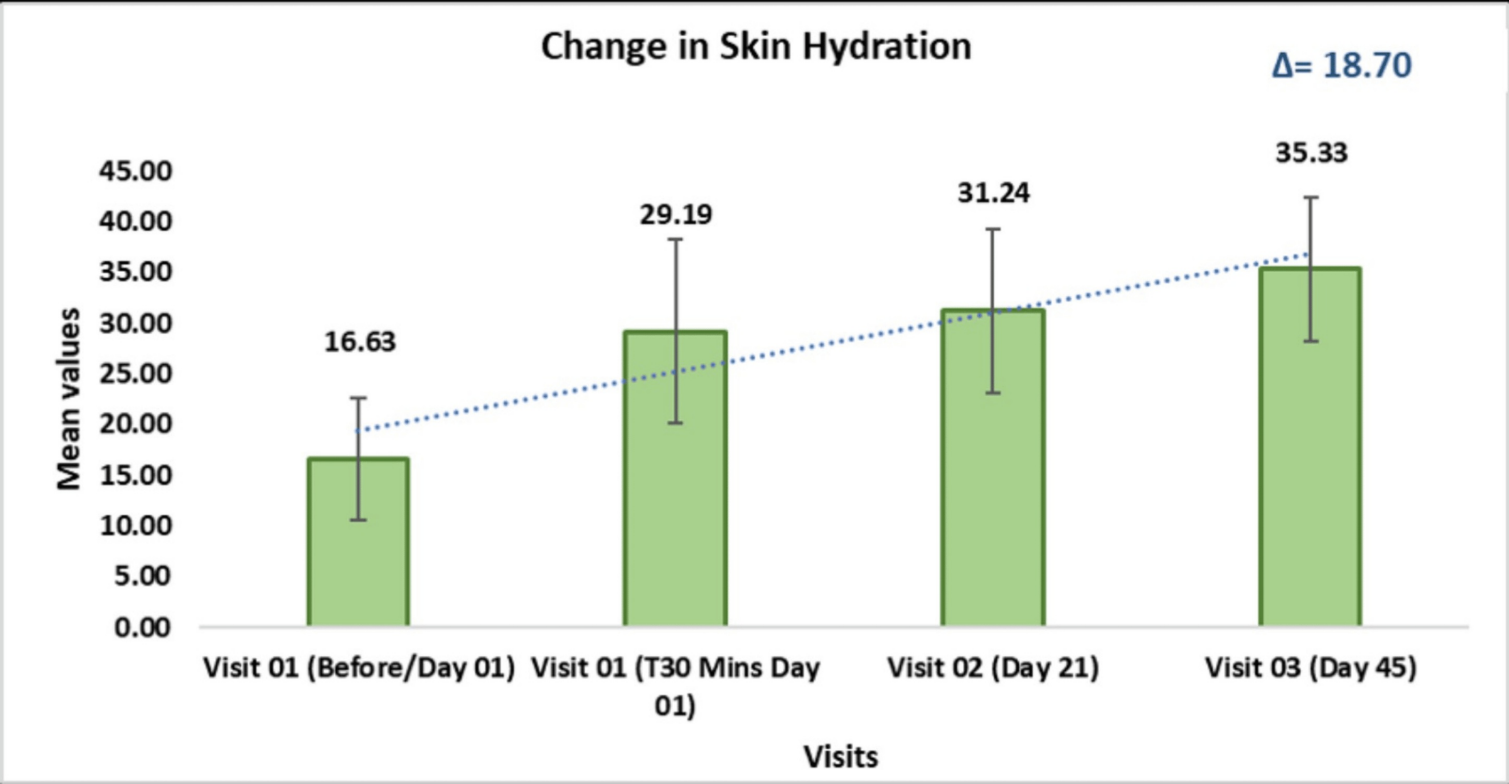
Assessment of skin hydration using the Corneometer® CM 825 Data are presented as mean ± standard deviation (SD). A p-value < 0.05 was considered statistically significant.

The skin hydration measured by the Corneometer® CM 825 demonstrated significant improvement throughout the study. From a baseline mean of 16.63 ± 6.03 on Day 01, hydration levels increased to 29.19 ± 9.03 at T30 minutes (p < 0.0001, t = 10.82), and further improved to 31.24 ± 8.07 on Day 21 (p < 0.0001, t = 12.84) and 35.33 ± 7.12 on Day 45 (p < 0.0001, t = 17.32). This represents a 1.76-fold improvement on Day 01, a 1.88-fold increase on Day 21, and a 2.12-fold enhancement by Day 45, highlighting that continuous use of the test kit significantly boosts skin hydration over time (Figure 2).

Skin Redness and Pigmentation Assessed by Mexameter® MX 18

**Figure 3:**
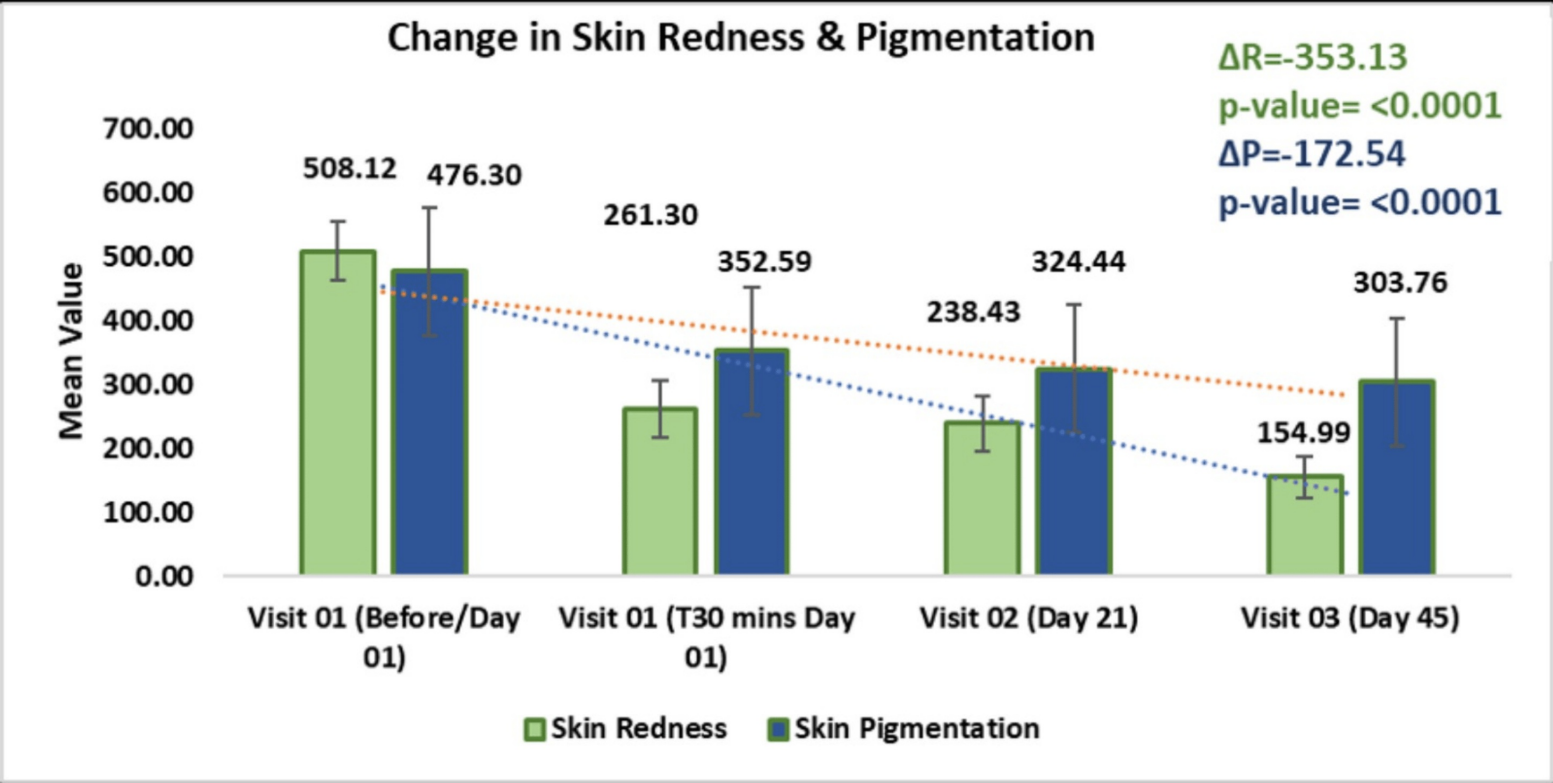
Assessment of skin redness and pigmentation using the Mexameter® MX 18 Data are presented as mean ± standard deviation (SD). A p-value < 0.05 was considered statistically significant.

Skin redness: The skin redness measured by the Mexameter® MX 18 showed a significant reduction over the study period. From a baseline mean of 508.12 ± 46.66 on Day 01, the redness decreased to 261.30 ± 43.98 at T30 minutes (p < 0.0001, t = -30.47), further reducing to 238.43 ± 43.09 on Day 21 (p < 0.0001, t = 25.47) and 154.99 ± 32.45 on Day 45 (p < 0.0001, t = 35.29). This represented a 1.94-fold reduction on Day 01, a 2.13-fold reduction on Day 21, and a 3.28-fold decrease by Day 45, indicating that continuous use of the test kit significantly reduces skin redness over time (Figure 3).

Skin pigmentation: The change in skin pigmentation assessed by the Mexameter® MX 18 showed a significant reduction in pigmentation over time. From a baseline mean of 467.30 ± 97.83 on Day 01, the mean value decreased to 352.59 ± 96.53 at T30 minutes (p < 0.0001, t = -26.67), further reducing to 324.44 ± 80.22 on Day 21 (p < 0.0001, t = -23.08) and 303.76 ± 84.63 on Day 45 (p < 0.0001, t = -22.04). This represented a 1.35-fold reduction on Day 01, a 1.47-fold reduction on Day 21, and a 1.57-fold reduction by Day 45, demonstrating that continuous use of the test kit significantly decreases skin pigmentation over time.

Skin Itchiness Assessed by the VAS Scale

**Figure 4:**
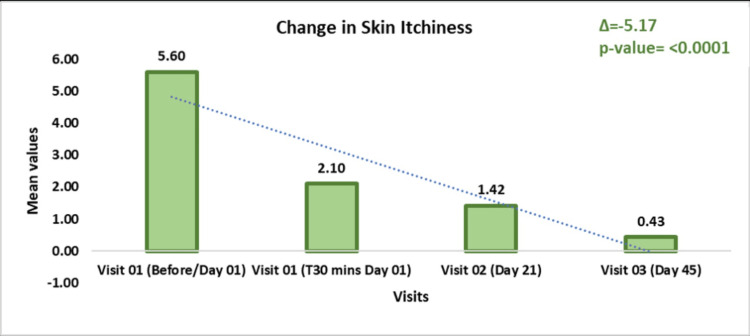
Assessment of skin itchiness using the Visual Analog Scale (VAS) Data are presented as mean ± standard deviation (SD). A p-value < 0.05 was considered statistically significant.

Skin itchiness, assessed by the VAS scale, showed a significant reduction over time. From a baseline mean of 5.60 ± 1.28 on Day 01, the mean decreased to 2.10 ± 1.45 at T30 minutes (p < 0.0001, t = -13.86), further dropping to 1.42 ± 1.18 on Day 21 (p < 0.0001, t = 17.00) and 0.43 ± 0.57 on Day 45 (p < 0.0001, t = -21.96). This represented a 2.67-fold reduction on Day 01, a 3.95-fold reduction on Day 21, and a 12.92-fold reduction by Day 45, indicating that continuous use of the test kit significantly alleviates skin itchiness over time (Figure 4).

Secondary endpoints

Smoothness Assessed by the VISIOSCAN® VC20 Plus

Skin smoothness, assessed by the VISIOSCAN® VC20 Plus, showed significant enhancement over time. From a baseline mean of 375.96 ± 70.68 on Day 01, the mean decreased to 188.47 ± 64.11 at T30 minutes (p < 0.0001, t = -13.46), further decreasing to 206.42 ± 57.66 on Day 21 (p < 0.0001, t = -14.48) and 166.36 ± 44.18 on Day 45 (p < 0.0001, t = -17.92). This represented a 1.99-fold improvement on Day 01, a 1.82-fold improvement on Day 21, and a 2.26-fold improvement by Day 45, indicating that continuous use of the test significantly improves skin smoothness over time.

Roughness Assessed by the VISIOSCAN® VC20 Plus

Skin roughness, assessed by the VISIOSCAN® VC20 Plus, showed a significant reduction over time. From a baseline mean of 1.39 ± 0.41 on Day 01, the mean increased to 2.22 ± 0.74 at T30 minutes (p < 0.0001, t = 7.07), further rising to 2.43 ± 0.92 on Day 21 (p < 0.0001, t = 5.95) and 3.19 ± 0.99 on Day 45 (p < 0.0001, t = 10.02). This represented a 1.59-fold reduction in roughness on Day 01, a 1.74-fold reduction on Day 21, and a 2.29-fold reduction by Day 45, demonstrating that continuous use of the test kit significantly decreases skin roughness over time.

Scaliness Assessed by the VISIOSCAN® VC20 Plus

Skin scaliness, assessed by the VISIOSCAN® VC20 Plus, showed a significant reduction over time. From a baseline mean of 3.94 ± 2.64 on Day 01, the mean decreased to 1.46 ± 1.95 at T30 minutes (p < 0.0001, t = -5.33), further decreasing to 1.42 ± 1.39 on Day 21 (p < 0.0001, t = -5.21) and 0.42 ± 0.67 on Day 45 (p < 0.0001, t = -7.93). This represented a 2.70-fold reduction in scaliness on Day 01, a 2.77-fold reduction on Day 21, and a 9.29-fold reduction by Day 45, indicating that continuous use of the test kit significantly decreases skin scaliness over time.

Treatment perception questionnaire

In the study, two subjects (6.67%) had previously used other products, while 23 subjects (93.33%) had not used any prior treatments. When evaluating the earlier test product, two (6.67%) subjects found it neither effective nor ineffective in reducing bumps and improving smoothness. For reducing dryness, roughness, redness, itchiness, and scaliness, one (3.33%) found the treatment neither effective nor ineffective, and one (3.33%) considered it slightly effective. Overall, one (3.33%) subject reported being neither satisfied nor dissatisfied with the test kit, while another one (3.33%) expressed slight satisfaction (Table 1).

**Table 1:** Treatment perception questionnaire - before test product use

Questionnaire item (before product usage)	Response/score	Count (%)
1. Subject has used any test product in the past for bumps treatment	Yes	2 (6.67)
	No	28 (93.33)
2. Earlier usage of the product name	Ponds	2 (6.67)
	Not Applicable (NA)	28 (93.33)
3. Earlier product effectiveness in reducing bumps on the skin	Score 5	2 (6.67)
	NA	28 (93.33)
4. Earlier product effectiveness in improving skin smoothness	Score 5	2 (6.67)
	NA	28 (93.33)
5. Earlier product effectiveness in reducing dryness, roughness, redness, itchiness, and scaliness	Score 5	1 (3.33)
	Score 6	1 (3.33)
	NA	28 (93.33)
6. Earlier product effectiveness in terms of overall satisfaction	Score 5	1 (3.33)
	Score 6	1 (3.33)
	NA	28 (93.33)

In the study, 30 (100%) subjects found the test kit effective in reducing bumps, with three (10%) rating it moderately effective, 22 (73.33%) very effective, and five (16.67%) extremely effective. Similarly, 30 (100%) subjects reported improvement in skin smoothness, with three (10%) rating it moderately effective, 12 (40%) very effective, and 15 (50%) extremely effective. For reducing dryness, roughness, and scaliness, three (10%) rated it moderately effective, 16 (53.33%) very effective, and 11 (36.67%) extremely effective. Regarding redness and itchiness, 23 (76.67%) found it very effective, and seven (23.33%) extremely effective. Overall, 100% of subjects were satisfied with the test kit, with 15 (50%) very satisfied and 15 (50%) extremely satisfied (Table 2).

**Table 2:** Treatment perception questionnaire - after test product use

Questionnaire item (after product usage)	Moderately effective	Very effective	Extremely effective
Reduction in bumps	3 (10.00%)	22 (73.33%)	5 (16.67%)
Improvement in smoothness	3 (10.00%)	12 (40.00%)	15 (50.00%)
Reduction in dryness, roughness, and scaliness	3 (10.00%)	16 (53.33%)	11 (36.67%)
Reduction in redness and itchiness	0 (0.00%)	23 (76.67%)	7 (23.33%)
Overall satisfaction	0 (0.00%)	15 (50.00%)	15 (50.00%)

Digital photographs

**Figure 5:**
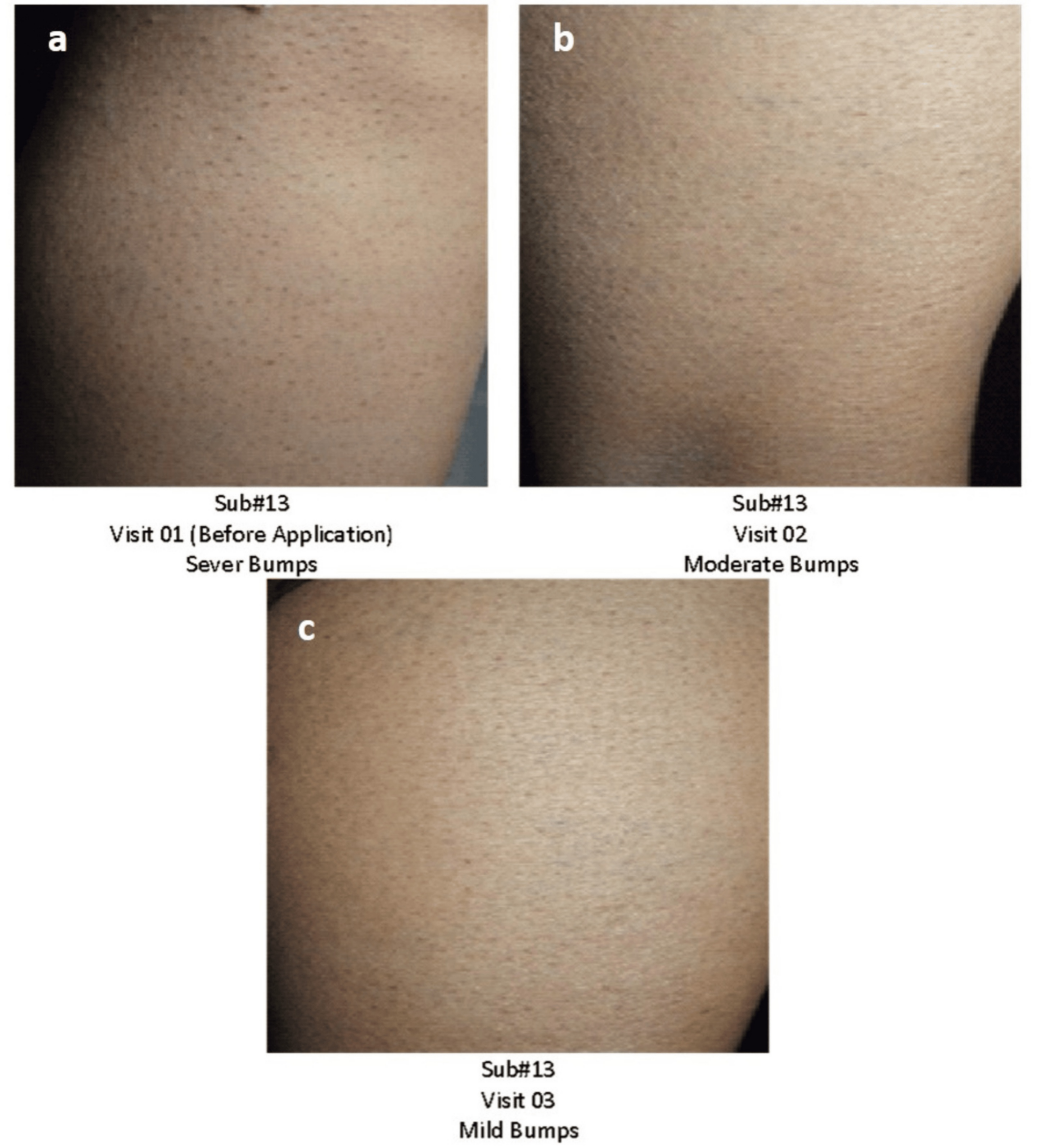
Digital photographs of Subject #13 (a) Bumps before usage of the test product; (b) moderate bumps at Day 21; (c) mild bumps at Day 45.

**Figure 6:**
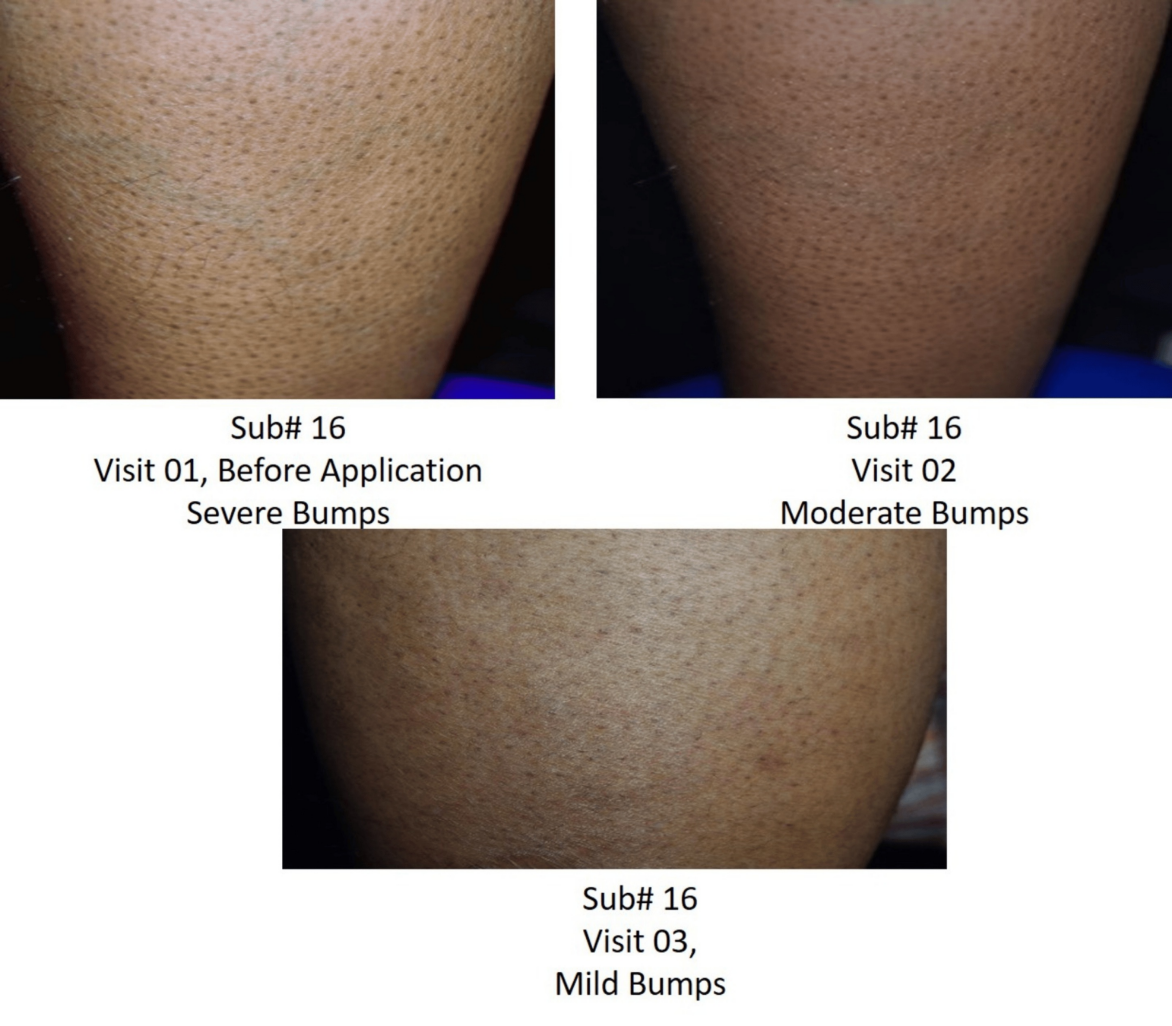
Digital photographs of Subject #16 (a) Bumps before usage of the test product; (b) moderate bumps at Day 21; (c) mild bumps at Day 45.

In the study, skin hydration, redness, and pigmentation were evaluated through a detailed photographic assessment. High-resolution images of the affected skin areas were captured under standardized conditions under the same light intensity and distance covered to ensure consistency across all subjects. These images were captured through a Nikon Digital Camera D3300 (Nikon Corporation, Tokyo, Japan) and then analyzed to assess the extent and severity of skin redness and pigmentation over time (Figures 5-6).

## Discussion

KP or strawberry legs is a benign skin condition marked by follicular hyperkeratosis. While it is typically asymptomatic, the appearance of KP can lead to psychosocial distress for patients. Although new treatments are emerging, further research is needed to evaluate their effectiveness [14]. KP is a papulosquamous disorder characterized by scaly papules and plaques, with an unclear etiology. It is believed to be inherited in an autosomal dominant pattern with variable penetrance. The most widely accepted theory proposes that the keratotic infundibular plug in KP results from abnormal keratinization of the follicular epithelium [15].

Hydroxy acids are commonly used in skin creams for their exfoliating and rejuvenating effects on photoaged skin. They also have antimicrobial, anti-inflammatory, and anticomedogenic properties, making them effective for treating acne [16].

The study investigated the impact of spirulina-based cosmetics on skin tone, hydration, hyperpigmentation, and fine lines in middle-aged women. After six weeks, the experimental group showed significant improvements in skin tone, brightness, and moisture, with reduced hyperpigmentation and erythema, suggesting spirulina-based cosmetics are more effective than standard skin care [17].

Several studies on AHA agents, such as glycolic acid and lactic acid, suggest that AHA has been used as a therapeutic agent to improve both the dermal and epidermal layers of the skin. These studies highlight the role of AHA in supporting dermal components and demonstrate its effectiveness in rejuvenating photo-damaged skin [18].

A phase 3, randomized, double-blind, vehicle-controlled trial was conducted to evaluate the efficacy and safety of 6% allantoin. Allantoin is a stable, soluble compound that demonstrates multiple wound-healing effects, including anti-inflammatory and antimicrobial properties, as well as promoting tissue formation and collagen deposition [19].

A randomized, controlled, double-blind, split-body study was conducted to evaluate the treatment effect of 7.5% urea cream compared to a basic moisturizing cream in patients. Both treatments significantly improved skin hydration, with urea cream showing superior results on the legs, where keratinization was most pronounced [20].

The Bumps Eraser Kit, consisting of a scrub and lotion, features spirulina in the scrub, which provides antioxidant and antimicrobial properties that neutralize free radicals and help protect the skin from oxidative stress and damage [21]. AHAs in the scrub aid in exfoliation [22]. The lotion contains Madhuca indica oil, which soothes roughness and provides antioxidant activity [23]; allantoin, which acts as a moisturizer to treat or prevent rough, scaly, and itchy skin [24]; and hydroxyethyl urea, which provides excellent moisturization. The scrub provides antioxidant activity while exfoliating the skin, enhancing smoothness. The lotion hydrates and nourishes the skin, supporting overall skin health. Together, the scrub and lotion help reduce scaliness, pigmentation, and itchiness, resulting in smoother skin.

The results clearly demonstrated significant improvements in skin smoothness, hydration, and a reduction in roughness and scaliness. Notably, there was a visible reduction in skin roughness, accompanied by increased smoothness. These changes highlight the product’s effectiveness in enhancing overall skin texture and appearance. The Test Product Bumps Eraser Kit was found to be both safe and effective for healthy adult females, with 100% of subjects reporting improvements in their skin. The key ingredients effectively address various skin concerns, improving texture, promoting smoother, clearer skin, and maintaining hydration.

A key limitation of the study is its open-label design, which introduces potential bias, and the lack of blinding or randomization affects the validity of the results. The small sample size and short 45-day duration limit the generalizability and long-term relevance of the findings. Future research should involve larger, more diverse populations, with longer follow-up periods and randomized controlled trials (RCTs) to strengthen the evidence. Additionally, considering lifestyle and environmental factors will help refine treatment strategies and improve their effectiveness.

## Conclusions

The ThriveCo Bump Eraser Kit, consisting of the exfoliating scrub and smoothing lotion, has demonstrated a cumulative effect, showing significant effectiveness and safety in treating common skin concerns such as bumpy skin, strawberry legs, and itchiness in adults. The synergistic action of its active ingredients improves skin hydration, texture, pigmentation, and irritation. The exfoliating scrub enhances smoothness and provides antioxidant benefits, while the nourishing lotion delivers deep hydration and soothes the skin. Together, these components effectively reduce scaliness, pigmentation, and itchiness, offering a comprehensive and reliable solution for achieving smoother, healthier skin. These products deliver measurable benefits in daily skincare by enhancing skin texture, alleviating scaliness and pigmentation, and improving the overall aesthetic appearance of the skin.
